# Effect of calcium on the interaction of *Acinetobacter baumannii* with human respiratory epithelial cells

**DOI:** 10.1186/s12866-019-1643-z

**Published:** 2019-11-27

**Authors:** Yi Chen, Tingjun Shao, Sanhua Fang, Ping Pan, Jiahui Jiang, Tongtong Cheng, Haitong Wan, Daojun Yu

**Affiliations:** 1 Hangzhou First People’s Hospital, Zhejiang Chinese Medical University, Hangzhou, China; 2 Department of Clinical Laboratory, Zhejiang Hospital, Hangzhou, China; 3 Zhejiang University School of Medicine, Hangzhou, China; 4 Zhejiang Chinese Medical University, Hangzhou, China

**Keywords:** *Acinetobacter baumannii*, Calcium; host-bacterial interaction, Infection

## Abstract

**Background:**

Investigating the factors that influence *Acinetobacter baumannii*(Ab) adhesion/invasion of host cells is important to understand its pathogenicity. Metal cations have been shown to play an important role in regulating the biofilm formation and increasing the virulence of Ab; however, the effect of calcium on host-bacterial interaction has yet to be clarified. Here, the dynamic process of the interaction between Ab and human respiratory epithelial cells and the effect of calcium on host-bacterial interaction were explored using microscopic imaging, quantitative PCR and real time cellular analysis (RTCA).

**Results:**

The concentration of calcium, multiplicity of infection and co-culture time were all demonstrated to have effects on host-bacterial interaction. A unique “double peak” phenomenon changed to a sharp “single peak” phenomenon during the process of Ab infection under the effect of calcium was observed in the time-dependent cell response profiles. Moreover, calcium can increase Ab adhesion/invasion of epithelial cells by regulating the expression of Ab-related genes (*ompA*, *bfmRS*, *abaI*).

**Conclusions:**

Effective control of calcium concentrations can provide new approaches for the prevention and treatment of multi-drug resistant Ab.

## Background

Over the last decades, *Acinetobacter baumannii*(Ab) has ranked among the most important Gram-negative nosocomial pathogens worldwide because of its increased rate of clinical isolation and the continuous emergence of multidrug-resistant strains [1, 2]. Ab is known for its prolonged survival in hospital settings, owing to its great capacity to adhere to abiotic or biotic surfaces (e.g., human respiratory epithelial cells) and its high tolerance to varied environmental conditions [3, 4]. Thus Ab is forecasted to become one of the biggest challenges to health care. The adhesion of Ab to epithelial cells is considered an essential first step in colonization and infection. Colonization or infection with Ab occurs mostly in critically ill patients and causes severe pneumonia or bloodstream infections that result in increased in morbidity and mortality in these patients, which is a troublesome problem for clinical diagnosis, treatment and prevention [1, 5, 6]. Moreover, it is important to emphasize that colonization with Ab is more common than infection, even in susceptible populations [5, 7–9]. Although the pathogenicity of Ab is generally low, bacterial colonization is the greatest risk factor for nosocomial infection. Once the balance between host and microbes tips towards infection, results are often severe and frequently lead to an increased probability of Ab hospital outbreaks. Therefore, the research on the control of Ab infection and colonization is of great practical significance and value.

In view of the seriousness of the antibiotic resistance and mass colonization by Ab on biotic surfaces, the pathogenicity of Ab has aroused widespread concern, especially in regard to the host-bacteria interaction and the process and molecular mechanism of Ab adhesion/invasion of human respiratory epithelial cells. Host-bacterial interaction is affected by many factors including biofilm formation and endotoxin production. In addition, biofilm formation represents an important factor associated with virulence and is affected by bacterial fimbriae, outer membrane proteins, adhesins, metal ions, quorum sensing, and complex regulatory networks (e.g., two-component regulatory systems), among others [10]. Investigating the factors affecting Ab adhesion/invasion of epithelial cells is important to understand its pathogenicity.

Metal cations have been shown to play an important role in regulating biofilm formation and differential expression of Ab-related genes, as well as in increasing the virulence of Ab and its ability to adhere to epithelial cells; however, the effects of calcium on host-bacterial interaction has not been elucidated [11, 12]. Studies have shown that bacterial infection can lead to a destabilization of the cellular calcium homeostasis and the activation of the calpain system ultimately triggering cell death, which suggests that changing the concentration of calcium in the environment may have a significant impact on the pathogenicity of Ab [13]. Lee et al. showed that bacterial attachment and biofilm formation on human respiratory epithelial cells and plastic surfaces were markedly reduced in the presence of the chelating agent EDTA (which effectively lowers the availability of metal cations such as Ca^2+^ and Mg^2+^) through the analysis of a group of multidrug-resistant Ab clinical isolates [14]. These data suggested that high concentrations of calcium may promote biofilm formation of Ab and enhance its adhesion to respiratory epithelial cells. However, this work did not specify the effect of calcium. It is important to explore the effect of calcium on host-bacterial interaction and to elucidate the functional mechanism, thus making it possible to take effective measures to control bacterial biofilm formation, adhesion and invasion and to ultimately provide new ideas for addressing the challenges of colonization and infection with multidrug-resistant Ab.

A label-free and noninvasive detection system (RTCA S16 system, ACEA Biosciences Inc.) based on dynamical and quantitative monitoring of cellular impedance in real time can produce specific time-dependent cell response profile (TCRPs) patterns. This approach can provide biological information related to cellular physiological function for the study of host-bacterial interaction [15]. Therefore, in this work, we developed microscopic imaging, quantitative PCR (qPCR) and TCRP methods for continuously monitoring the interaction between Ab and human respiratory epithelial cells and the effect of calcium on host-bacterial interaction. Our research can be used to study the calcium-mediated signaling pathway in human respiratory epithelial cells infected with Ab, which provides a basis for the study of the pathogenicity of Ab [16].

## Results

### Optimum multiplicity of infection (MOI) of Ab to human respiratory epithelial cells

**Fig. 1:**
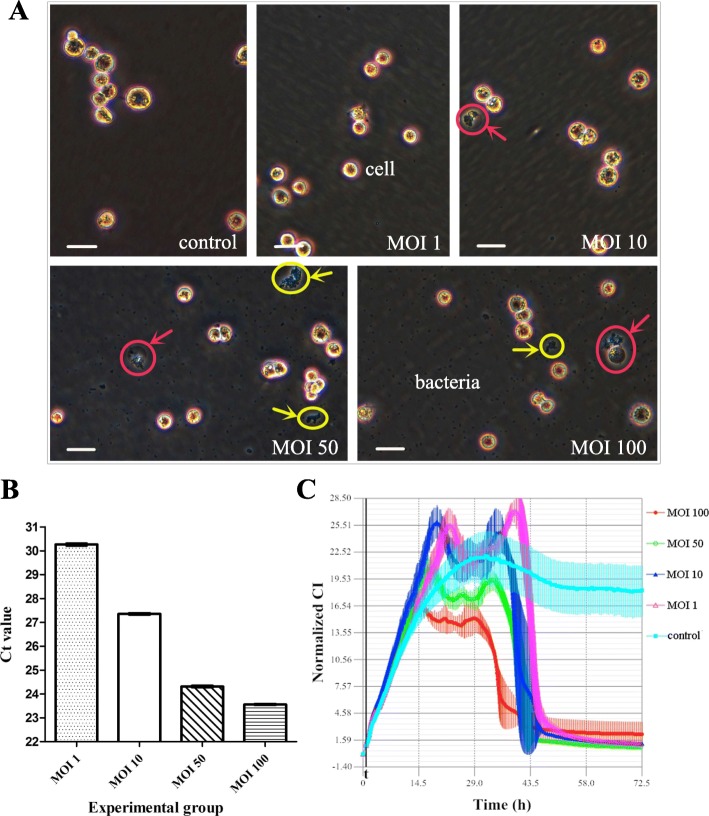
Analysis of optimum MOI of Ab to human respiratory epithelial cells. Control: 0.45% NaCl. **a** Microscopy observations of the effect of Ab on epithelial cells (2 × 10^5^ cells per well) at different MOIs **(**co-cultured for 4 h). Scale bar = 30 μm. Red arrows: bubble-like (denucleated) dead cells with bacterial adhesion visible around (especially in the MOI 50 and MOI 100 experimental groups); Yellow arrows: the phenomenon of bacterial aggregation. **b** The qPCR results indicating Ab invasion of epithelial cells (including strong adhesion) at different MOIs (host cells and bacteria were co-cultured for 4 h and 1 × 10^5^ cells were isolated). The differences in the Ct values (MOI 100 group < MOI 50 group < MOI 10 group < MOI 1 group) among all the groups were statistically significant, as determined by the SNK test (*P* < 0.05). The differences between the MOI 1 and MOI 10 groups and the MOI 10 and MOI 50 groups were greater than between the MOI 50 and MOI 100 groups. The higher the MOI was, the greater the Ab invasion of epithelial cells. **c** Ab infection TCRPs of epithelial cells (20,000 cells per well) at different MOIs. t: point at which bacteria or NaCl were added (after background readings for the E-Plate were obtained and the plate was incubated at room temperature for 30 min). The “double peak” phenomenon emerged during Ab infection. The smaller the MOI was, the later and higher the peak CI was, the more significant the phenomenon was. Representative curves are an average of three replicate wells

The effects of Ab on the morphology and proliferation of human respiratory epithelial cells at different MOIs and co-culture time points were determined by inverted microscopy and are shown in Table 1 and Fig. 1A. After host-bacterial co-culture for 2 h, therewere relatively few bacteria and almost no adhesion to epithelial cells. The differences between the groups were also relatively small (especially in the control group and the MOI 1 and MOI 10 experimental groups). After co-culture for approximately 4 h, the differences betweenthe groups became more obvious. With the increase of MOI, the effect of Ab on the epithelial cells was also increased (the differences between the MOI 50 and MOI 100 experimental groups were not obvious). Additionally, compared with other co-culture time points, the changes in cell morphology and proliferation at 4 h of co-culture were more typical. Therefore, the co-culture time point of 4 h was selected as the appropriate time point for subsequent studies. In addition, at 6–8 h of co-culture, the quantity of bacteria was relatively large, which was not suitable for further research and analysis.

**Table 1:** Microscopy observations of the effect of Ab on epithelial cells at different MOIs

Co-culture time point	Control group ^a^	Experimental group ^b^			
		MOI 1	MOI 10	MOI 50	MOI 100
2 h	+ / -	+ (−) / ±(−)	+ (−) / ++ (−)	+ (+) / & (+)	+ (+) / & (+)
4 h	++ / -	++ (−) / + (−)	++ (±) / +++ (−)	++ (++) / && (++)	++ (++) / && (++)
6 h	+++ / -	+++ (−) / ++ (−)	+++ (+) / ++++ (−)	+++ (+++) / &&& (+++)	+++ (+++) / &&& (+++)
8 h	++++ / -	++++ (−) / ++ (−)	++++ (+) / ++++ (±)	+++ (+++) / &&&& (+++)	+++ (+++) / &&&& (+++)

Control: 0.45% NaCl

^a^: epithelial cells / bacteria; ^b^: epithelial cells (bubble-like dead cells) / bacteria (bacterial aggregation phenomenon)

+: indicated the degree (the epithelial cells were expressed as the situation of adherent growth, bacteria were expressed as quantity, bubble-like dead cells and bacterial aggregation phenomenon were expressed as visibility); &: indicated the degree > ++++; −: indicated none

For example: + / - indicated that a few of epithelial cells adhered to plates without bacteria; +++ (+++) / &&& (+++) indicated that the bacterial number increased and the bacterial aggregation phenomenon became more obvious compared to 4 h, a lot of epithelial cells adhered to plates and the bubble-like dead cells with bacterial adhesion visible

Bacterial invasion (including strong adhesion) to epithelial cells at different MOIs was determined by qPCR. The host cells and bacteria were co-cultured for 4 h and 1 × 10^5^ cells were isolated for qPCR. The qPCR results (threshold cycles, Ct values) are shown in Fig. 1B. With the increase of MOI, the invasion of Ab into human respiratory epithelial cells increased gradually (the smaller the Ct value was, the more bacterial invasion occurred). There was no invasion in the control group. The difference in the Ct values among all the experimental groups was statistically significant (*P* < 0.05). MOI 100 group (Ct values: 23.56 ± 0.04) < the MOI 50 group (24.31 ± 0.05) < the MOI 10 group (27.36 ± 0.05) < the MOI 1 group (30.26 ± 0.11). The difference in the Ct values between the MOI 10 and MOI 50 groups or between the MOI 1 and MOI 10 groups was approximately 3, while the difference between the MOI 50 and MOI 100 groups was relatively small (an approximately 0.8 difference in the Ct values).

TCRPs were determined by real time cellular analysis. The cell index (CI) induced by Ab infection increased at first and then decreased (Fig. 1C). With the decrease of MOI, the time to reach the peak CI was gradually delayed and the peak CI was also gradually increased. A unique “double peak” phenomenon emerged in the process of infection (the smaller the MOI was, the more obvious the phenomenon was). The bacterial concentration of the MOI 50 experimental group was most appropriate, and the phenomenon characteristics were more typical than that in MOI 100 group. Therefore, the bacterial concentration (1 × 10^8^ CFU/ml) corresponding to the MOI 50 was suitable for subsequent studies based on the above results.

### Effect of Ab on different incubation states of human respiratory epithelial cells

**Fig. 2:**
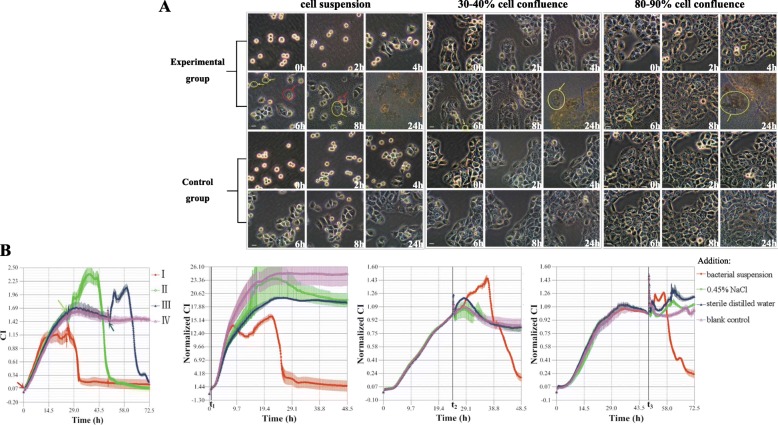
The effect of Ab on human respiratory epithelial cells in different incubation states. **a** Microscopy observation of the effect of Ab on epithelial cells (4 × 10^5^ cells per well) in different incubation states. Scale bar = 30 μm. Experimental group: addition of bacterial suspension; Control group: addition of 0.45% NaCl; Red arrows: bubble-like (denucleated) dead cells; Yellow arrows: the phenomenon of bacterial aggregation; Blue arrows: massive cell death. **b** Ab infection TCRPs of epithelial cells (20,000 cells per well) in different incubation states. I: addition of bacterial suspension (red arrow) at cell suspension (0 h); II: addition of bacterial suspension (green arrow) at 30–40% cell confluence (after 24 h); III: addition of bacterial suspension (blue arrow) at 80–90% cell confluence (after 48 h); IV: blank control (without intervention); t: treatment time point; t_1_: cell suspension (0 h); t_2_: 30–40% cell confluence (after 24 h); t_3_: 80–90% cell confluence (after 48 h). Ab infection caused the CI to rise and fall. A unique “double peak” phenomenon emerged during Ab infection. The addition of 0.45% NaCl or sterile distilled water had no significant effect on the CI

Ab had an almost identical effect on the different incubation states of epithelial cells (Fig. 2A). In the early stages of host-bacterial co-culture, epithelial cells can adhere to the plastic surface, although bubble-like dead cells (nuclear pyknosis, cell swelling anddissolution, Additional file 1: Figure S1) can be seen (after approximately 4 h). However, with the prolongation of co-culture time and the increase of the number of bacteria, the changes in cell morphology and proliferation, as well as the bacterial aggregation phenomenon became more typical (full field of bacteria after 8 h). It was observed that Ab had an effect on the adherent growth of epithelial cells. After 24 h, the bacteria dominated the entire cell culture dish, while the host cells were all dead.

The dynamic processes (as determined based on the TCRPs) of the interaction between Ab and epithelial cells in different states of cell incubation were also similar (Fig. 2B). For instance, the initial Ab infection had little effect on cell growth, and the “double peak” phenomenon emerged during Ab infection (this was not typical when the CI was about to enter the platform period). Almost identical cell growth curves were observed for the 0.45% sodium chloride solution (NaCl) and sterile distilled water groups. Compared with the untreated group, the slight decrease or increase of CI in 0.45% NaCl and sterile distilled water groups may have been associated with the dilution of nutrients or metabolites in the culture medium. Therefore, the effect of 0.45% NaCl in the bacterial suspension on the adherent growth of epithelial cells could be considered negligible.

### Effect of calcium on Ab proliferation and biofilm formation

**Fig. 3:**
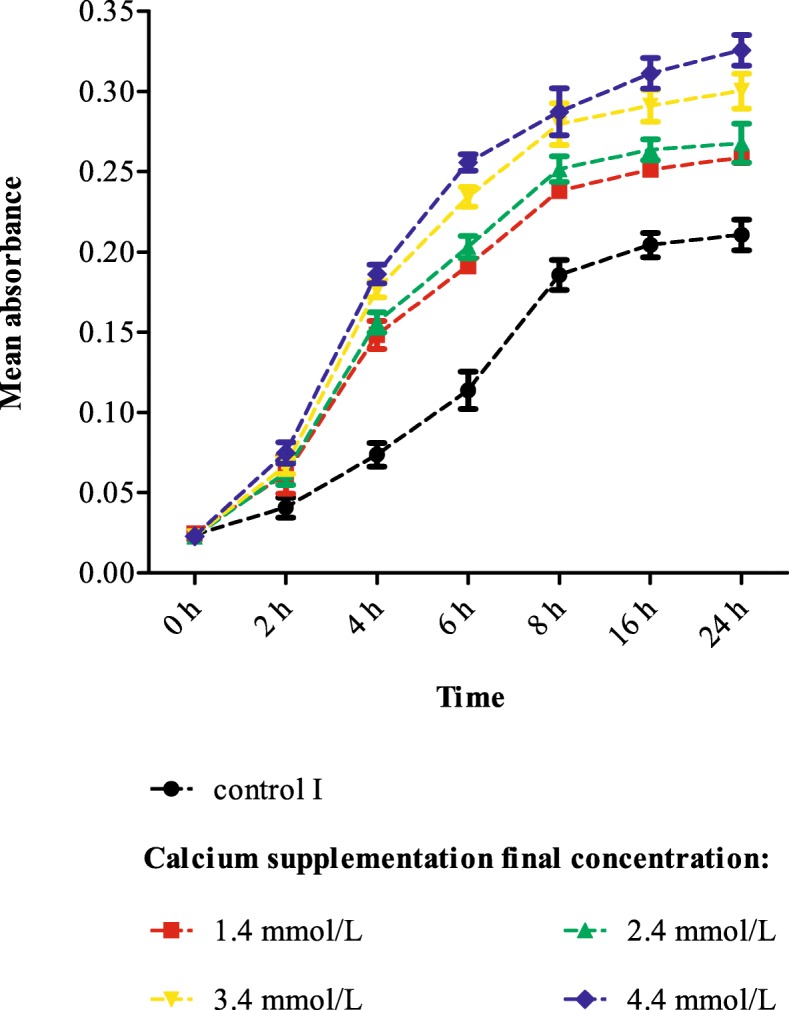
The effect of calcium on Ab proliferation (growth curves). Control I: calcium final concentration was 0 mmol/L (with EDTA treatment).Calcium can promote the proliferation of Ab

**Fig. 4:**
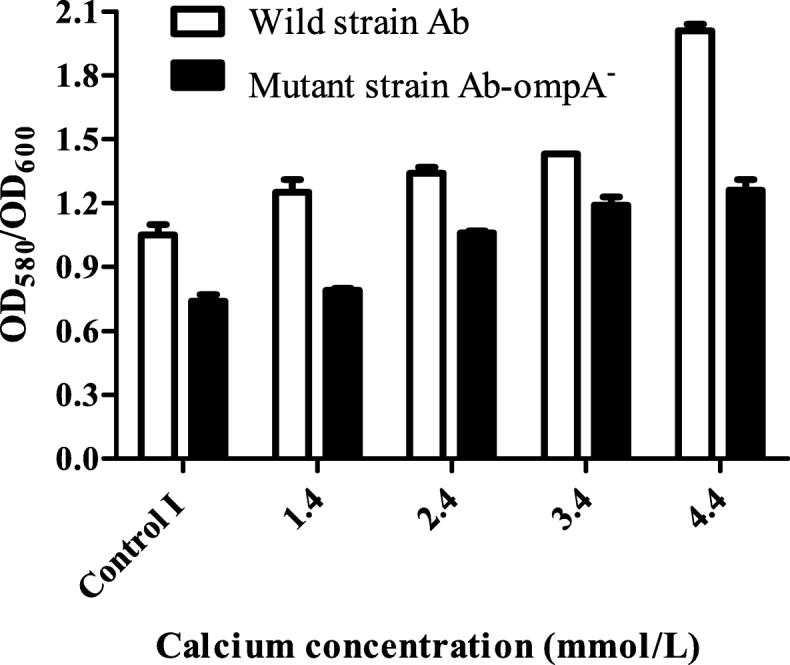
Biofilm assay results. Control I: calcium final concentration was 0 mmol/L (with EDTA treatment). Calcium may promote Ab biofilm formation, and *ompA* has a positive effect on biofilm formation

As shown in Fig. 3, we found that high concentrations of calcium could contribute to the proliferation of Ab and that this effect was more pronounced with time. There was a difference in biofilm formation between Ab and Ab-ompA^−^ isolates (*P* = 0.00), indicating *ompA* had a significant effect on biofilm formation. Ab deficient in *ompA*could reduce biofilm formation. Additionally, the results showed that calcium concentration was effective as a treatment factor (*P* = 0.00), calcium may promote biofilm formation. The bacterial biofilm formation was more obvious with the increase of calcium concentration (Fig. 4).

### Effect of calcium on the morphology and proliferation of human respiratory epithelial cells

It was difficult to distinguish differences between the groups using inverted microscopy, so we next used TCRPs to evaluate the effects of calcium on the proliferation of human respiratory epithelial cells. The CI of epithelial cells increased significantly with both increasing calcium concentrations (≤4.4 mmol/L) and culture times (≤24 h)(Additional file 2: Figure S2). The CI values of each group under different calcium concentrations and culture times (0 h, 2 h, 4 h, 6 h, 8 h, 12 h and 24 h) were compared by multivariate ANOVA with repeated measures and the SNK test. The results showed that time was effective as a factor (*P* = 0.00), meaning that CI changes with time. There was a positive interaction between time and treatment (calcium concentrations) factors (*P* = 0.00). Exogenous calcium supplementation can promote the growth of human respiratory epithelial cells (Additional file 3: Table S1). The higher the calcium concentration was (≤4.4 mmol/L) and the longer the culture time was (≤24 h), the more significant the promoting effect on CI values was.

### Effect of calcium on host-bacterial interaction

The role of calcium in bacterial proliferation was the same as has been described above. With the increase of co-culture time and calcium concentrations, the bacterial aggregation phenomenon (biofilm formation) became more obvious (Additional file 4: Figure S3). After 8 h, there was an obvious increase in the quantity of bacteria. After approximately 24 h, the bacteria encompassed the full field, while massive numbers of host cells were dead. It is known that Ab can have effects on the adherent growth of epithelial cells; however, the effects of calcium on the interactions between Ab and epithelial cells could not be determined using an inverted microscopy.

**Fig. 5:**
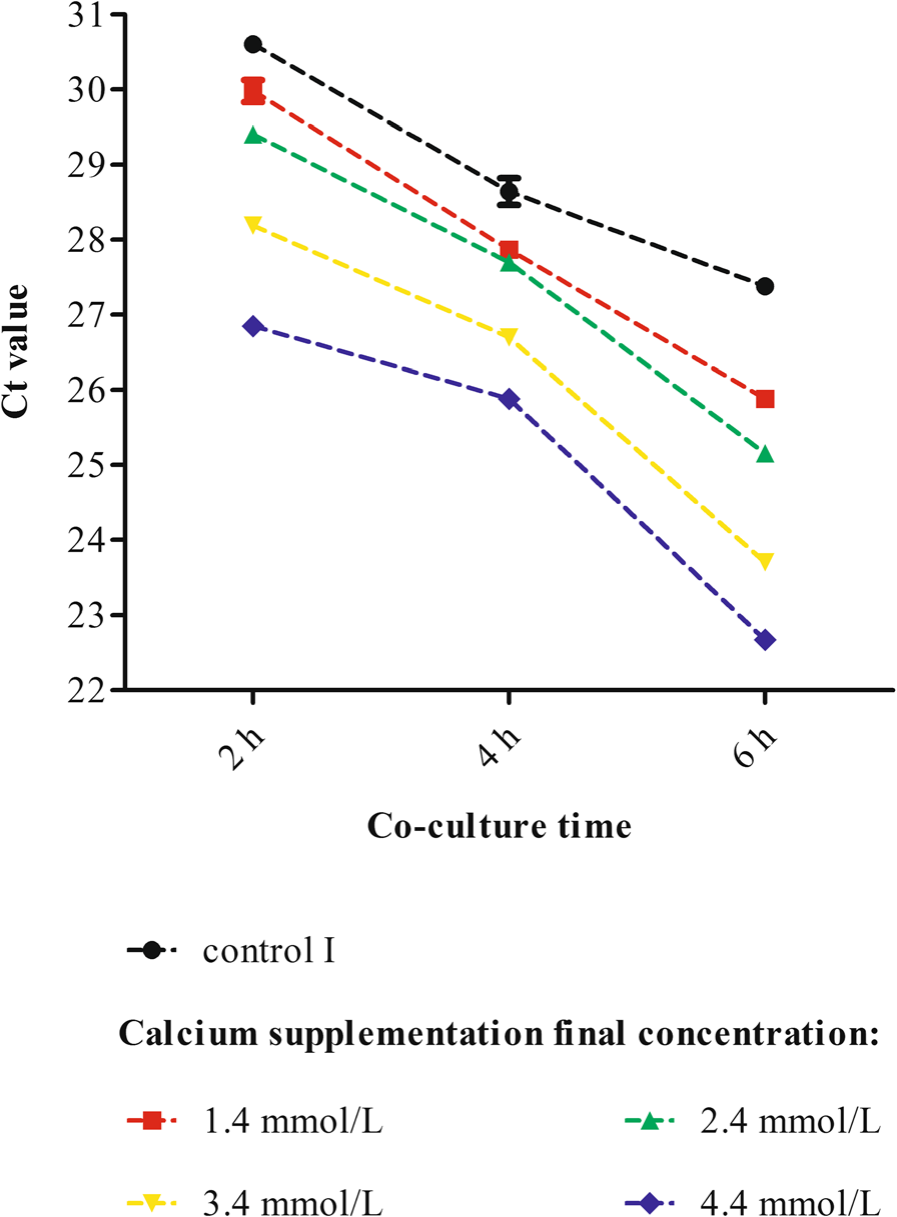
qPCR results of calcium effect on host-bacterial interaction. Ab and human respiratory epithelial cells were co-cultured in calcium-supplemented medium for 2 h, 4 h and 6 h, and 1 × 10^5^cells were isolated for qPCR detection. Control I: calcium final concentration was 0 mmol/L (with EDTA treatment). Calcium had positive effect on the interaction between Ab and epithelial cells. With the increase of calcium concentrations and prolonged co-culture times, the amount of Ab invasion into epithelial cells increased (the smaller the Ct value, the more bacterial invasion to cells)

The host and bacteria were co-cultured in calcium-supplemented medium for 2 h, 4 h and 6 h. The effects of calcium on bacterial invasion (including strong adhesion) of epithelial cells (1 × 10^5^ cells as the standard) were determined using qPCR and shown in Fig. 5. The results of control group II were negative. The Ct values were compared by univariate ANOVA with repeated measures and the SNK test. Time as a factor was significant with a *P* = 0.00, which meant that the amount of Ab invading (including tightly adhered to) epithelial cells changed over time. Moreover, treatment (calcium concentrations) as a factor was effective (*P* = 0.00), meaning that the amount of Ab invading (including tightly adhered to) epithelial cells varied according to grouping, and this difference among the groups was statistically significant (*P* < 0.05). There was a positive correlation between the time and treatment factors (*P* = 0.00). The role of time as a factor varied from group to group. As a result, the higher the calcium concentration was and the longer the co-culture time was, the more frequently Ab invaded epithelial cells.

**Fig. 6:**
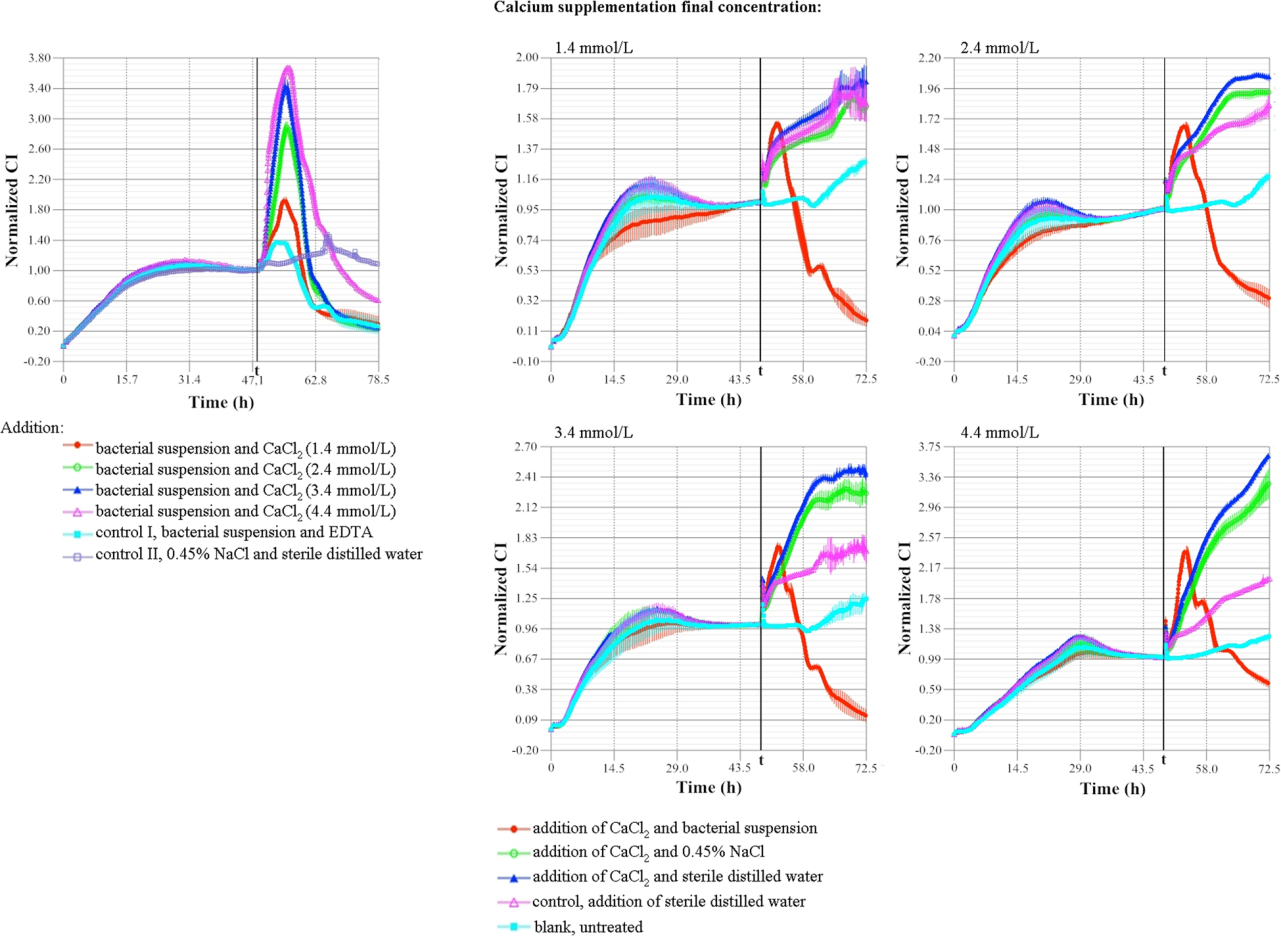
The effect of calcium on Ab infection TCRPs of human respiratory epithelial cells. A total of 20,000 cells per well were seeded into E-plates. t: treatment time point (48 h, 80–90% cell confluence). With increased calcium concentrations (bacteria-infected cells), the CI increased or decreased faster and reached a higher peak value. A sharp “single peak” phenomenon occurred during the infection

TCRPs showed that either initial Ab infection or the action of calcium could induce the increase of CI. When increasing calcium concentration in host and bacteria culture medium (≤ 4.4 mmol/L), the faster the CI rose, the higher the peak CI was (6-8 h), and the more significant cell growth stimulation was. By contrast, the CI declined rapidly with prolonged host-bacterial interactions (the higher the calcium concentration was, the faster the CI declined). Meanwhile, a sharp “single peak” phenomenon occurred in the process of infection (Fig. 6).

### Effect of calcium on the expression of Ab-related genes

**Fig. 7:**
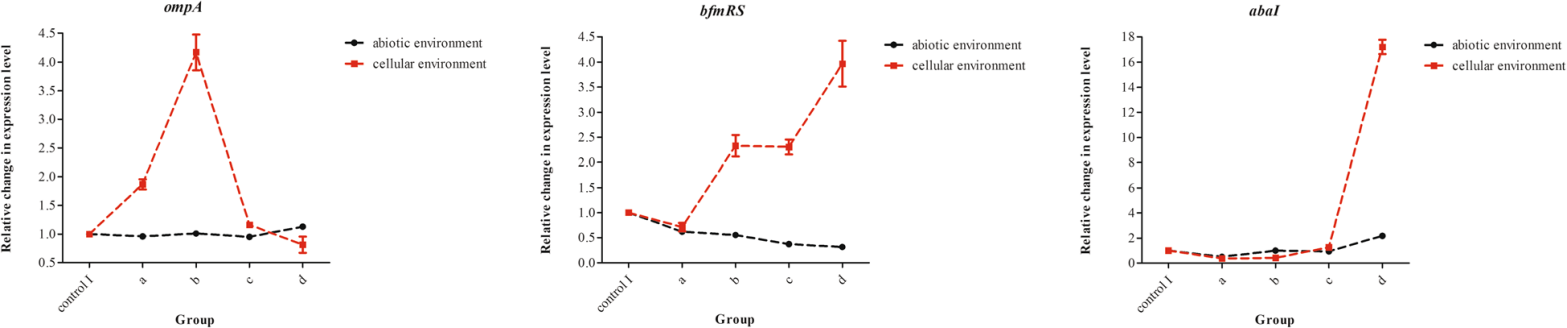
Effect of calcium on the expression of Ab-related genes. The calcium supplementation final concentrations were as follows: Group a, 1.4 mmol/L; Group b, 2.4 mmol/L; Group c, 3.4 mmol/L; Group d, 4.4 mmol/L; Group control I: calcium final concentration was 0 mmol/L (with EDTA treatment); Abiotic environment: bacteria only were cultured in plates containing RPMI 1640 medium; Cellular environment: bacteria were cultured in epithelial cell covered plates (80–90% cell confluence) containing RPMI 1640 medium. The *recA* gene was used as an internal reference. The relative changes of Ab related gene expression between the experimental groups and control I group were calculated by the 2^-△△Ct^ method

Calcium can affect the expression of Ab-related genes. The*recA* gene was used as an internal reference control. Both negative controls (I and II) had no amplification. Relative changes in the expression levels of target genes (*ompA*, *bfmRS*and*abaI*) between experimental groups and control I group were calculated by the 2^-△△Ct^ method. The trends were different between the abiotic and cellular environments that the bacteria were cultured in (Fig. 7).

There was no significant difference in the expression level of *ompA* among the groups cultured in the abiotic environment (*P* > 0.05), whereas expression of this gene showed significant differences in the cellular environment (*P* < 0.05). In the cellular environment, relative changes in expression levels between group c and control I, as well as between group d and control I were small (*P* > 0.05); however, *ompA* expression in group b was approximately 4-fold higher than that of the control I group.

In abiotic environment, there were significant differences in the expression of*bfmRS* between the experimental groups and the control I group (*P* < 0.05). With the increase of calcium concentration in the culture medium, *bfmRS* expression in the experimental groups showed a decreasing trend; its expression in group d was approximately 0.31-fold higher than that of the control I group. In the cellular environment there was no significant difference in the expression level between group a and the control I group (*P* > 0.05). Contrary to the expression trend in the abiotic environment, expression level of groups b and c were approximately 2.3-fold higher than that of the control I group, while that of group d was approximately 4-fold higher.

In the abiotic environment, the expression level of *abaI* in group b, c and control group I was similar (*P* > 0.05). The expression of *abaI* in group a was approximately 0.5-fold higher than that of the control I group, while its expression in group d was approximately 2-fold higher. In the cellular environment, the *abaI* expression in groups a and b was approximately 40% higher than that of the control I group, while that in group d was about 17 times higher than that in control group I.

## Discussion

Ab infection and colonization co-exist, mainly causing respiratory infections (such as ventilator-associated pneumonia) [17] that seriously endanger human life and quality of life and result in a major economic burden [18]. Elucidating the molecular mechanism of the interaction between Ab and host cells is of great significance for further understanding the pathogenic mechanism of this bacteria and proposing new prevention and treatment strategies.

Based on the normal blood calcium concentration of 2.25–2.75 mmol/L,the concentration of calcium in the media used in these experiments was controlled within 1.4–4.4 mmol/L to simulate the environment of the body. Our study found that exogenous calcium supplementation can promote the proliferation of Ab and the adherent growth of human respiratory epithelial cells, as well as induce differential expression of Ab-related genes. In addition, calcium also played an important role in host-bacterial interaction, promoting Ab adhesion/invasion of human respiratory epithelial cells and thereby increasing the degree of bacterial infection in the host cells. The higher the calcium concentration is (especially in the case of high calcium) and the longer the culture duration, the more severe the degree of host cells bacterial infection is. Calcium may affect the host-bacterial interaction through several factors.

RTCA detection is an important technique that can reflect changes of cell morphology (including size, shape, stretching, etc.), number and adhesion. Compared with traditional endpoint detection, RTCA has the advantages of non-invasive and high accurate, as well as providing real-time monitoring, complete TCRPs, and easy operation. It is widely used in cytology research, such as cell migration and invasion assays, cytotoxicity tests, gene regulation and cell-microenvironment interactions [15, 19–21]. Therefore, the obtained TCRPs can provide better information on the effect of calcium on host-bacterial interaction, which complements our microscopic observations. Interestingly, RTCA detection in the present study found unique “double peak” (calcium-free, Figs. 1c and 2b) and sharp “single peak” (calcium-containing, Fig. 6) phenomena during bacterial infection of epithelial cells. The sharp “single peak” may indicate that calcium stimulation promotes rapid cell growth (observed peak) at the initial stage of bacterial infection. When the number of bacteria reached the critical point, calcium in turn enhanced bacterial adhesion/invasion of epithelial cells, thereby rapidly decreasing the cell index (CI). The mechanism of the “double peak” phenomenon needs to be further studied and analyzed. Possible mechanisms include that cell death was caused by bacteria and cells stimulating, adapting and interacting with each other. Specifically, the bacteria were added to the cell culture environment as exogenous foreign bodies, which stimulated the proliferation of the cells in the initial stage of infection (the appearance of the first peak), but then the bacteria inhibited host cell proliferation due to its own rapid proliferation (the decrease in the first peak). With prolonged host-bacterial interaction, the cells became tolerant to the bacterial inhibition (adaptation) and continued to proliferate (the emergence of the second peak), after which the two competed with one another in the nutrient-rich environment. Additionally, the increasing toxic effects of the bacteria on the epithelial cells resulted in a rapid reduction in the CI (the decrease in the second peak).

Ab-ompA, a highly conserved outer membrane protein, is also an important virulence factor that plays an important role in bacterial infection (which is also supported by our research) and causes an up-regulation of epithelial cellular immune response signaling pathway [16, 17]. It has been shown that Ab-ompA secretes and transmits virulence factors through outer membrane vesicles. Translocation of ompA-containing vesicles into host cells can result in host cell apoptosis, whereas ompA mutants fail to induce cell death [22]. Thus, blocking or inhibiting the expression of Ab-ompA will greatly reduce Ab adhesion/invasion of host cells [23]. Our studies showed that compared with abiotic environments, Ab-ompA expression is obviously changed in cellular environments. Increased calcium content (especially at 2.4 mmol/L) may contribute to the expression of Ab-ompA, which is consistent with our finding that calcium can aggravate bacterial adhesion/invasion of host cells, while other factors may be involved under high calcium conditions. We found that Ab-ompA expression only changes significantly during host-bacterial interaction indicating that ompA is a virulence factor. Additionally, changing the calcium concentration of the environment may have a significant impact on Ab pathogenicity [13]. *OmpA* has a positive effect on biofilm formation was also proved in our experiment. Based on the above findings, the increase of calcium concentration may enhance the virulence of bacteria and promote the expression of *ompA* to some extent. Therefore, if calcium concentration is kept at the lower level of the normal range (2.2–2.4 mmol/L), it may be possible to prevent Ab invasion caused by the up-regulation of *ompA* expression. We suggest that for patients infected with *Acinetobacter baumannii*, it is necessary to regularly monitor and strictly control the concentration of serum calcium in order to prevent the deterioration of patients with *Acinetobacter baumannii* infection.

Ab-bfmRS is a key factor in the survival of Ab in the environment and is comprised of a sensor kinase (bfmS) and a response regulator (bfmR). The inactivation of bfmRS not only reduces biofilm formation but also leads to the loss of bacterial adhesion to eukaryotic cells [24]. Interestingly, the composition of the culture medium and the interaction of Ab with abiotic surfaces play a significant role when the BfmRS system is not expressed [12]. In addition, environmental signals are important for the bfmRS regulatory system and biofilm formation, and are involved in inducing the bacterial morphology responsible for interacting with abiotic surfaces [12]. These observations may explain why exogenous calcium supplementation in cellular and abiotic environments has completely different effects on*bfmRS* expression in our research. In abiotic environments, calcium inhibits the expression of *bfmRS*, which was negatively correlated, while in the cellular environment, calcium promotes*bfmRS* expression.This is consistent with the fact that calcium may promote biofilm formation and increase Ab adhesion/invasion. Calcium may be an environmental signaling molecule affecting the expression of Ab-bfmRS. Therefore, increasing the concentration of calcium may promote the formation of biofilm and enhance the bacterial adhesion/invasion of epithelial cells. As Ab-bfmRS expression is controlledby many factors, how calcium causes a down-regulation of *bfmS* expression in abiotic environments remains to be further studied.

The protein encoded by *abaI* is a very important self-inducible synthase of Ab that produces distinct acyl-homoserine lactone (AHL) signals that can up-regulate the expression of *abaI* through a positive feedback loop to promote the secretion of AHLs and biofilm maturation [25]. Our study found that calcium has a similar effect on Ab-abaI expression in the cellular and abiotic environments. Low concentrations of calcium (< 2.4 mmol/L) may inhibit *abaI* expression, while high concentrations of calcium tend to promote its expression. The change in Ab-abaI expression with high concentrations of calcium in the cellular environment was more significant than in the abiotic environment. Moreover, it is known that calcium may promote biofilm formation. These data suggest that bacterial biofilm formation may be reduced by controlling calcium concentrations (2.2–2.4 mmol/L), which may reduce Ab colonization or infection capacities in the environment.

*Acinetobacter baumannii*used in this paper was isolated from hospitalized patients and resistant to a variety of antimicrobial agents. At present, because the drug resistance rate of *Acinetobacter baumannii* is very high, and the clinical strain research can reflect the real clinical situation and has strong practicability, clinical multidrug-resistant strains were used to investigate the effect of calcium on the interaction between clinical strains and respiratory epithelial cells. However, there are many reports on the interaction between epithelial cells and *Acinetobacter baumannii* standard strains using either ATCC 19606 or ATCC 17978. The lack of experiments with standard strains is the shortcoming of this paper, so we have provided antimicrobial susceptibility testing results and genome sequencing results about the clinical multidrug-resistant isolate to make the experimental data comparable.

## Conclusions

Since the standard strain of Ab was not used in the study, the consistency between the results of this paper and previous reports needs to be further verified. According to the results of this experiment, we have come to the following preliminary conclusions. First, with the increase of MOI, Ab adhesion/invasion of human respiratory epithelial cells was gradually increased. Second, in the initial stage of infection, low concentrations of Ab have no significant effect on cell growth, and a unique “double peak” phenomenon emerged in the process of infection. Third, calcium may promote Ab biofilm formation, and *ompA* has a positive effect on biofilm formation; calcium may promote the proliferation of Ab and the adherent growth of human respiratory epithelial cells; furthermore, it can increase the adhesion/invasion of Ab to epithelial cells by regulating the expression of Ab-related genes (*ompA*, *bfmRS*, *abaI*). Lastly, calcium also has an impact on the interaction between Ab and human respiratory epithelial cells, and stimulates cell growth more significantly in the early stage of infection, resulting in a sharp “single peak” phenomenon. The higher the calcium concentration is and the longer the co-culture time is, the more serious the host cells were infected by bacteria. Based on the results of these experiments and the related literature [13, 14], it can be speculated that controlling calcium concentrations may play a role in the prevention and treatment of multi-drug resistant Ab colonization or infection.

## Methods

### Cells and bacteria

The human respiratory epithelial cells (HPAEpiC,Cat. No. 3200) were purchased from ScienCell Research Laboratories, Inc. (San Diego, California) and maintained in RPMI 1640 medium containing 10% fetal bovine serum and 100 U/ml penicillin/streptomycin (GIBCO) at 37 °C and 5% CO_2_. The use of HPAEpiC cells can better reflect the colonization or infection of *Acinetobacter baumannii* in human respiratory tract and is close to the human environment. The clinical isolate of multidrug-resistant Ab (Additional file 5: Table S2) was originally collected from the sputum of patient hospitalized in Hangzhou First People’s Hospital, China. After identification of genome sequencing (Sangon Biotech Co., Ltd., Shanghai; the SRA accession: PRJNA523637), the bacteria were inoculated to blood agar plates for 18–20 h at 37 °C, and then single colonies were picked to suspend in 0.45% sodium chloride solution for preparing bacterial suspension. The bacteria per ml was calculated based on 1 McF = 2 × 10^8^ CFU/ml.

### Host-bacterial co-culture

#### At different MOIs

The range of bacterial MOIs was selected according to previous studies [15]. A 1 ml aliquot of HPAEpiC (2 × 10^5^ cells/ml) was seeded into six-well plates containing RPMI 1640 medium. Immediately, 100 μl of different concentrations of bacteria (2 × 10^6^, 2 × 10^7^, 1 × 10^8^ and 2 × 10^8^ CFU/ml, corresponding to the MOIs 1, 10, 50 and 100, respectively) were inoculated into each well, and 0.45% NaCl was used as the normal control. The effects of Ab on the morphology and proliferation of epithelial cells at different MOIs and co-culture time points (2 h, 4 h, 6 h and 8 h) were observed by an IX70 bright field inverted microscopy (Olympus Optical Co., Ltd., Japan) at a magnification of 30×. Meanwhile, we identified the live and dead epithelial cells according to the LIVE-DEAD viability/cytotoxicity assay kit instructions (Life Technologies, Grand Island, NY, USA) and detected using an IX71 inverted microscopy with Nomarski optics (Olympus Optical Co., Ltd., Japan). Based on these observations, a suitable time period for the host-bacterial co-culture was selected for subsequent experiments.

The liquid in each well of the six-well plates was carefully discarded at the end of the indicated host-bacterial co-culture time period. Before digestion with trypsin solution for 1–2 min at 37 °C, the cells were washed once with phosphate buffer saline (PBS). The cells were then harvested by centrifugation (100×g, 5 min) and washed twice with PBS. Finally, bacterialDNA was extracted from 1 × 10^5^ cells based on procedures described by Chen et al [26]. qPCR was performed with SYBR Premix Ex Taq II (TaKaRa Bio Inc., Shiga, Japan) on an ABI 7500 real-time PCR instrument (Applied Biosystems, United States) according to the manufacturer’s instructions. Each test (50-μl volume) was performed in triplicate. Sterile distilled water served as a negative control, and bacterialDNA served as a positive control. Primer sequences for the target gene (*ompA*, outer membrane protein A) were as follows: 5′-CACAGATAACACTGGTCCACG-3′ and 5′-GAATACACGACGGTTCATAGC-3′. The changes in Ab invasion of epithelial cells (including strong adhesion) at different MOIs were anaylsed by comparing the Ct values obtained.

Detailed RTCA (real time cellular analysis) experimental procedures have been previously described [15, 19, 20]. Briefly, 50 μl of medium was added to 16-well E-plates (specific cell culture plates for RTCA) to obtain background readings, which were followed by the addition of 100 μl of a cell suspension (2 × 10^5^ cells/ml). After the E-Plate was incubated at room temperature for 30 min, and 10 μl of different concentrations of bacterial suspension or 0.45% NaCl was added to the wells containing cells. The E-Plates were placed onto the reader in the incubator for continuous recording of the CI. The cells were monitored every 5 min for 72 h to obtain TCRPs. The data were collected from three multiple-well duplicates and presented as the CI normalized (CI = 1.00) to the last time point before intervention. Additionally, a suitable bacterial concentration was selected for subsequent experiments based on the above results.

#### In different states of cell incubation

The purpose of this experiment was to observe the effect of *Acinetobacter baumannii* on cell proliferation under different cell culture conditions and cell adherent growth conditions. In other words, what kind of cell incubation (cell suspension, cell partial adherent fusion, cell complete adherent fusion) is more suitable to add bacterial suspension to cell petri dish (including cell and cell culture medium) to study host-bacterial interaction. This experiment was divided into three groups of different cell incubation states.

Cell suspension: the purpose of this experimental group was to observe the effect on the adhesion of Ab to host cell under initial suspension state of cell suspension added to six-well plates (0 h); 30–40% cell confluence: the purpose of this experimental group was to observe the effect of *Acinetobacter baumannii* on epithelial cells without growth space restriction (The cells have been cultured for 24 h); 80–90% cell confluence: the purpose of this experimental group was to observe the effect of *Acinetobacter baumannii* on epithelial cells under the cell incubation state of 80–90% cell confluency after 48 h of cell culture, this state was close to the human environment.

A total of 2 ml of cells (2 × 10^5^ cells/ml) were seeded into six-well plates containing RPMI 1640 medium. 200 μl of the indicated concentration of bacteria was added to wells containing cells in different incubation states (cell suspension, 30–40% cell confluence and 80–90% cell confluence) and 0.45% NaCl was used as the normal control. An inverted microscopy was used to observe the dynamic process of the interaction between Ab and epithelial cells at different states of cell incubation (2 h, 4 h, 6 h, 8 h and 24 h).

RTCA experimental procedures were as described above. A total of 10 μl of the above mentioned bacterial suspensions, 0.45% NaCl or sterile distilled water was added to wells containing cells at different incubation states (cell suspension, 30–40% cell confluence and 80–90% cell confluence). Wells without intervention were used as the blank control. The interaction between Ab and human respiratory epithelial cells were evaluated based on the TCRP results and microscopic imaging.

### Effect of calcium on host-bacterial interaction

#### Ab growth assays

A total of 400 μl of each of the indicated concentrations of bacteria and different concentrations (12 mmol/L, 24 mmol/L, 36 mmol/L and 48 mmol/L) of a calcium chloride solution (CaCl_2_) were added to wells containing 2 ml of RPMI 1640 medium. A 400 μl aliquot of each of the bacterial suspensions and EDTA solution (5 mmol/L) was used as the normal control I (taking into account the presence of calcium in RPMI, the final calcium concentrations in experimental groups were 1.4 mmol/L, 2.4 mmol/L, 3.4 mmol/L and 4.4 mmol/L, respectively, and the normal control I was 0 mmol/L). The effect of different concentrations of calcium and culture times (2 h, 4 h, 6 h, 8 h, 16 h and 24 h) on the proliferation of Ab were measured at 600 nm using a microplate reader (Sunrise, Tecan, Switzerland). Each experiment was performed in triplicate.

#### Experiments on the differential expression of Ab-related genes

The sequences of Ab-related genes were selected according to previous studies [12, 16, 27, 28]. Specifically, the *recA* (internal reference), *ompA*(outer membrane protein A),*bfmRS*(two-component regulatory system) and*abaI* (autoinducer synthase) sequences were downloaded from GenBank. The primers (Table 2) for reverse transcription quantitative PCR (RT-qPCR) were designed using the Primer Premiers 5.0 software, selected based on a BLAST sequence comparison, and synthesized by Life Technology (Shanghai, China). After incubation for the indicated time period described above, all bacteria were collected and total RNA was isolated from each group using an AxyPrep Miniprep Kit (Corning Inc., NY, USA) according to the manufacturer’s instructions. The concentration and purity (OD_260_/OD_280_ of approximately 2.0) of the RNA samples were measured using a NanoDrop 2000 spectrophotometer (Thermo Fisher Scientific, Wilmington, USA). RT-qPCR was performed with the One Step SYBR PrimeScript PLUS RT-PCR Kit (TaKaRa) on an ABI 7500 real-time PCR instrument according to the manufacturer’s instructions. Each test (50 μl volume, 20 ng of RNA template) was performed in triplicate. RNase free distilled water served as negative control I and templates treated with RNase served as negative control II. The differential expression of Ab-related genes (*ompA*, *bfmRS*and *abaI*) under different calcium concentrations was anaylsed by comparing the resulting Ct values.

**Table 2:** Primers for RT-qPCR

Gene ID ^a^	Primers	Sequences (5′–3′)	Product length (bp)
Reference gene			
AF251469.1	recA-F recA-R	ACGCCCTAGACCCTCAATAT AGAGTCACCCATCTCACCTTC	197
Target gene			
AY485227.1	ompA-F ompA-R	CACAGATAACACTGGTCCACG GAATACACGACGGTTCATAGC	190
AY838282.1	bfmRS-F bfmRS-R	AACAAAGTTCGGATTACGGG TCATCTAAACGGGCAAAGG	128
EU334497.1	abaI-F abaI-R	CTATTCCCTGCTCACCAGA CCCGCAGCACGTAATAAAC	208

^a^: GenBank

#### Ab biofilm assays

Mutant strains of Ab deficient in *ompA* (Ab-ompA^−^) were constructed and modified according to the conjugative transfer and parental conjugation methods [29, 30]. Briefly, a modified pMO130-Tel^R^ with the fragments corresponding to the regions up and downstream of the *ompA* gene generated from genome of Abwas constructed. The resultant plasmid was then transformed into the*E.coli* S17–1 λ pir competent cells. Then, trans-conjugation was performed between *E.coli* S17–1 λ pir donor strain and Ab recipient strain to transfer and integrate pMO130-Tel^R^*-ompA (Up/Down)* into the chromosome of Ab.Donor and recipient strains were plated onto LB agar containing tellurite (30 mg/L) and gentamicin (25 mg/L) and incubated at 37 °C overnight. 0.45 mol/L catechol solution was sprayed on the surface of the plate, and yellow clones were picked for PCR identification. The second selection was then performed by incubating Ab on 10% sucrose with salt-free LB agar plates, stayed overnight at 37 °C to identify sucrose-resistant sensitive clones, then analyzed by PCR and sequencing to confirm that the target gene was excised, resulting in an unmarked in-frame deletion. A single clone with no *ompA* sequence was saved as Ab-ompA^−^. Ab and Ab-ompA^−^ isolates were added separately to 96-well plates containing RPMI 1640 medium of different calcium concentrations (range from 0 to 4.4 mmol/L). The biofilm formation was measured based on procedures outlined by Selasiet al [31]. with some modifications. After 24 h, the liquid in each well was carefully discarded and washed three times with PBS. Then, the plates were air-dried and stained with crystal violet (0.1% v/v) for 20 min. Finally, the plates were washed three times with PBS, air-dried and decolorized with ethanol (95% v/v) for 20 min. The turbidity was measured at 580 nm using the Tecan Sunrise microplate reader. Additionally, the turbidity was also measured at 600 nm before biofilm mass staining to compensate for bacterial growth differences. Biofilm formation was quantified by calculating the ratio of OD_580_/OD_600_. Each experiment was performed in triplicate.

#### Experiments on the morphology and proliferation of epithelial cells

A 2 ml cell suspension (2 × 10^5^ cells/ml) was seeded into six-well plates and allowed to attach and grow for 48 h to reach the platform stage (80–90% cell confluence) before the addition of a 400 μl aliquot of each of sterile distilled water and different concentrations of CaCl_2_. Additionally, a 400 μl aliquot of each of sterile distilled water and EDTA was used as the normal control. The effects of different concentrations of calcium and culture times (2 h, 4 h, 6 h, 8 h and 24 h) on the morphology and proliferation of epithelial cells were observed by inverted microscopy.

RTCA experimental procedures were as described above. After incubation for 48 h, cells were treated with a 20 μl aliquot of each of sterile distilled water and different concentrations of CaCl_2_ or a 20 μl aliquot of each of sterile distilled water and EDTA. A well without intervention was used as a blank control. The effects of calcium on the morphology and proliferation of epithelial cells were evaluated based on the TCRP results and microscopic imaging.

#### Experiments on host-bacteria interactions

A 2 ml cell suspension (2 × 10^5^ cells/ml) was seeded into wells and allowed to attach and grow for 48 h before the addition of a 400 μl aliquot of each of the indicated bacterial concentrations and different concentrations of CaCl_2_. Additionally, a 400 μl aliquot of each of the bacterial suspensions and EDTA was used as the normal control I. The effect of Ab on the morphology and proliferation of epithelial cells with different calcium concentrations and co-culture times (2 h, 4 h, 6 h, 8 h and 24 h) were observed by inverted microscopy.

According to the procedures described above, total bacterial RNA was isolated from each group and RT-qPCR was performed after host cells and bacteria were co-cultured for the indicated time period. The expression of Ab-related genes in the biotic environment under different calcium concentrations was anaylsed by comparing the Ct values obtained.

In addition, a 400 μl aliquot of each of sterile distilled water and 0.45% NaCl was used as the normal control II. All the liquid in the wells was carefully discarded after a 2 h, 4 h or 6 h incubation, as described above. Before digestion, the cells were washed three times with PBS. Other procedures, including bacterial DNA extraction and qPCR were carried out as described above. The changes of Ab invasion to epithelial cells (including strong adhesion) under different calcium concentrations and co-culture times was anaylsed by comparing the Ct values obtained.

RTCA experimental procedures were as described above. After the cells (20,000 cells per well) were incubated for 48 h, the two part experiment was carried out as follows:

1. A 20 μl aliquot of each of the abovementioned bacterial suspensions and different concentrations of CaCl_2_ was added to the wells; the bacterial suspensions and EDTA was used as the normal control I; sterile distilled water and 0.45% NaCl was used as the normal control II.
2. A 20 μl aliquot of each of different concentrations of CaCl_2_ and the bacterial suspension/0.45% NaCl/sterile distilled water was added to the wells; a total of 20 μl of sterile distilled water was added as the normal control. Wells without intervention were used as the blank control.

The effect of calcium on the host-bacterial interactions was evaluated based on the TCRP results, qPCR and microscopic imaging.

### Statistical analysis

The data are presented as the means ± SD. One-way analysis of variance (ANOVA) and the Student-Newman-Keuls (SNK) test were applied to compare and analyse the changes in Ct values at different MOIs and the effects of calcium on the expression of Ab-related genes. Two-way ANOVA and the SNK test were applied to analyse the effects of calcium on biofilm formation of different Ab strains. The effects of different calcium concentrations and culture times on the CI of epithelial cells were analysed using multivariate ANOVA with repeated measures and the SNK test. Univariate ANOVA with repeated measures and the SNK test were used to compare and analyse the effects of different calcium concentrations and co-culture times on Ab invasion (including strong adhesion) of epithelial cells (Ct values). *P* < 0.05 was considered to be statistically significant.

## Supplementary information

**Additional file 1: Figure. S1.** Co-culture of *Acinetobacter baumannii* (Ab) and epithelial cells.

**Additional file 2: Figure. S2.** Effect of calcium on TCRPs of human respiratory epithelial cells.

**Additional file 3: Table S1.** Effect of calcium concentrations on the CI of epithelial cells.

**Additional file 4: Figure. S3.** Microscopy observations of the effect of calcium on host-bacterial interactions.

**Additional file 5: Table S2.** Results of antimicrobial susceptibility testing of Ab used in this study.

## Data Availability

All data generated or analysed during this study are included in this published article.

## Abbreviations

Ab: *Acinetobacter baumanii*
AHL: N-acyl homoserine lactone
ANOVA: Analysis of variance
CI: Cell index
Ct: Cycle threshold
MOI: Multiplicity of infection
qPCR: quantitative PCR
RTCA: Real time cellular analysis
RT-qPCR,: Reverse transcription quantitative PCR
SNK: Student-Newman-Keuls
TCRP: Time-dependent cell response profile
