# Supplementary material for: Effect of calcium on the interaction of Acinetobacter baumannii with human respiratory epithelial cells
Source: BMC Microbiol. 2019 Nov 27;19:264. doi: 10.1186/s12866-019-1643-z (PMC6880639; doi:10.1186/s12866-019-1643-z)
Supplement: Supplementary file 1 — Additional file 1: Figure. S1. Co-culture of Acinetobacter baumannii (Ab) and epithelial cells. [file 12866_2019_1643_MOESM1_ESM.doc]

Additional file 1

A B


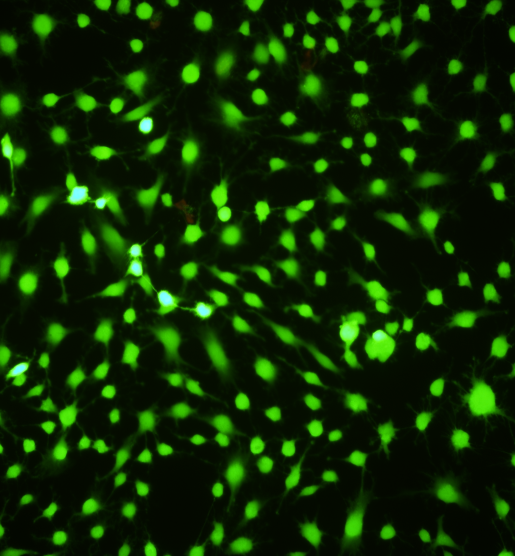

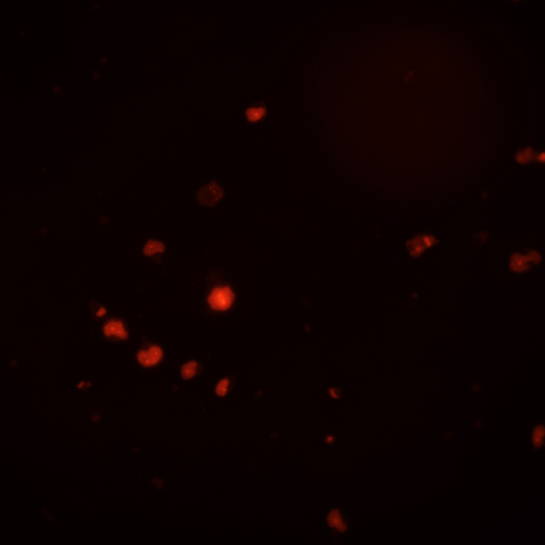


Fig. S1. **Co-culture of *Acinetobacter baumannii* (Ab) and epithelial cells.**

Co-culture of *Acinetobacter baumannii* (Ab) and epithelial cells were stained with LIVE-DEAD viability/cytotoxicity assay kit and the images were captured using Olympus florescence microscope (200×). (A) Epithelial cells can adhere to the plastic surface without Ab and stained with live (green). (B) Bubble-like dead cells (nuclear pyknosis, cell swelling and dissolution) can be seen after approximately 4 h of co-culture and stained with dead (red).
