# Supplementary material for: Effect of calcium on the interaction of Acinetobacter baumannii with human respiratory epithelial cells
Source: BMC Microbiol. 2019 Nov 27;19:264. doi: 10.1186/s12866-019-1643-z (PMC6880639; doi:10.1186/s12866-019-1643-z)
Supplement: Supplementary file 2 — Additional file 2: Figure. S2. Effect of calcium on TCRPs of human respiratory epithelial cells. [file 12866_2019_1643_MOESM2_ESM.doc]

Additional file 2


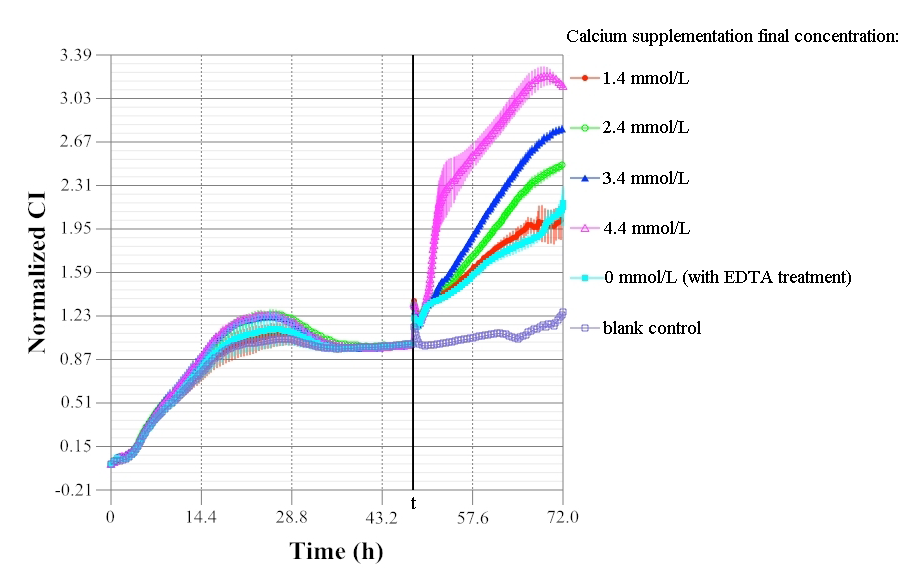


Fig. S2. **Effect of calcium on TCRPs of human respiratory epithelial cells.**

t: treatment time point (48 h, 80-90% cell confluence).

Calcium may promote the adherent growth of human respiratory epithelial cells.
