# Supplementary material for: Effect of calcium on the interaction of Acinetobacter baumannii with human respiratory epithelial cells
Source: BMC Microbiol. 2019 Nov 27;19:264. doi: 10.1186/s12866-019-1643-z (PMC6880639; doi:10.1186/s12866-019-1643-z)
Supplement: Supplementary file 3 — Additional file 3: Table S1. Effect of calcium concentrations on the CI of epithelial cells. [file 12866_2019_1643_MOESM3_ESM.doc]

Additional file 3

Table S1. **Effect of calcium concentrations on the CI of epithelial cells.**

| Group | 0 h | 2 h | 4 h | 6 h | 8 h | 12 h | 24 h |
| --- | --- | --- | --- | --- | --- | --- | --- |
| Control | 1.00±0.00 | 1.29±0.02 | 1.36±0.01 | 1.42±0.02 | 1.49±0.02 | 1.68±0.03 | 2.18±0.13 |
| a | 1.00±0.00 | 1.31±0.01 | 1.40±0.01 | 1.46±0.01 | 1.54±0.01 | 1.74±0.03 | 2.05±0.17 |
| b | 1.00±0.00 | 1.31±0.00 | 1.44±0.02 | 1.51±0.01 | 1.63±0.02 | 1.88±0.03 | 2.48±0.03 |
| c | 1.00±0.00 | 1.29±0.02 | 1.46±0.03 | 1.60±0.01 | 1.77±0.02 | 2.10±0.03 | 2.78±0.03 |
| d | 1.00±0.00 | 1.34±0.02 | 2.11±0.19 | 2.33±0.21 | 2.46±0.13 | 2.71±0.10 | 3.11±0.03 |

Control group: the calcium final concentration was 0 mmol/L (with EDTA treatment). The calcium supplementation final concentrations were as follows: Group a, 1.4 mmol/L; Group b, 2.4 mmol/L; Group c, 3.4 mmol/L; Group d, 4.4 mmol/L.

After 12 h, the experimental groups b, c and d were greater than the control group in the cell index (CI), and the differences among the groups were statistically significant (*P* < 0.05, SNK test). While no significant difference was observed between the experimental group a and the control group (*P* > 0.05).
