# Supplementary material for: Effect of calcium on the interaction of Acinetobacter baumannii with human respiratory epithelial cells
Source: BMC Microbiol. 2019 Nov 27;19:264. doi: 10.1186/s12866-019-1643-z (PMC6880639; doi:10.1186/s12866-019-1643-z)
Supplement: Supplementary file 4 — Additional file 4: Figure. S3. Microscopy observations of the effect of calcium on host-bacterial interactions. [file 12866_2019_1643_MOESM4_ESM.doc]

Additional file 4


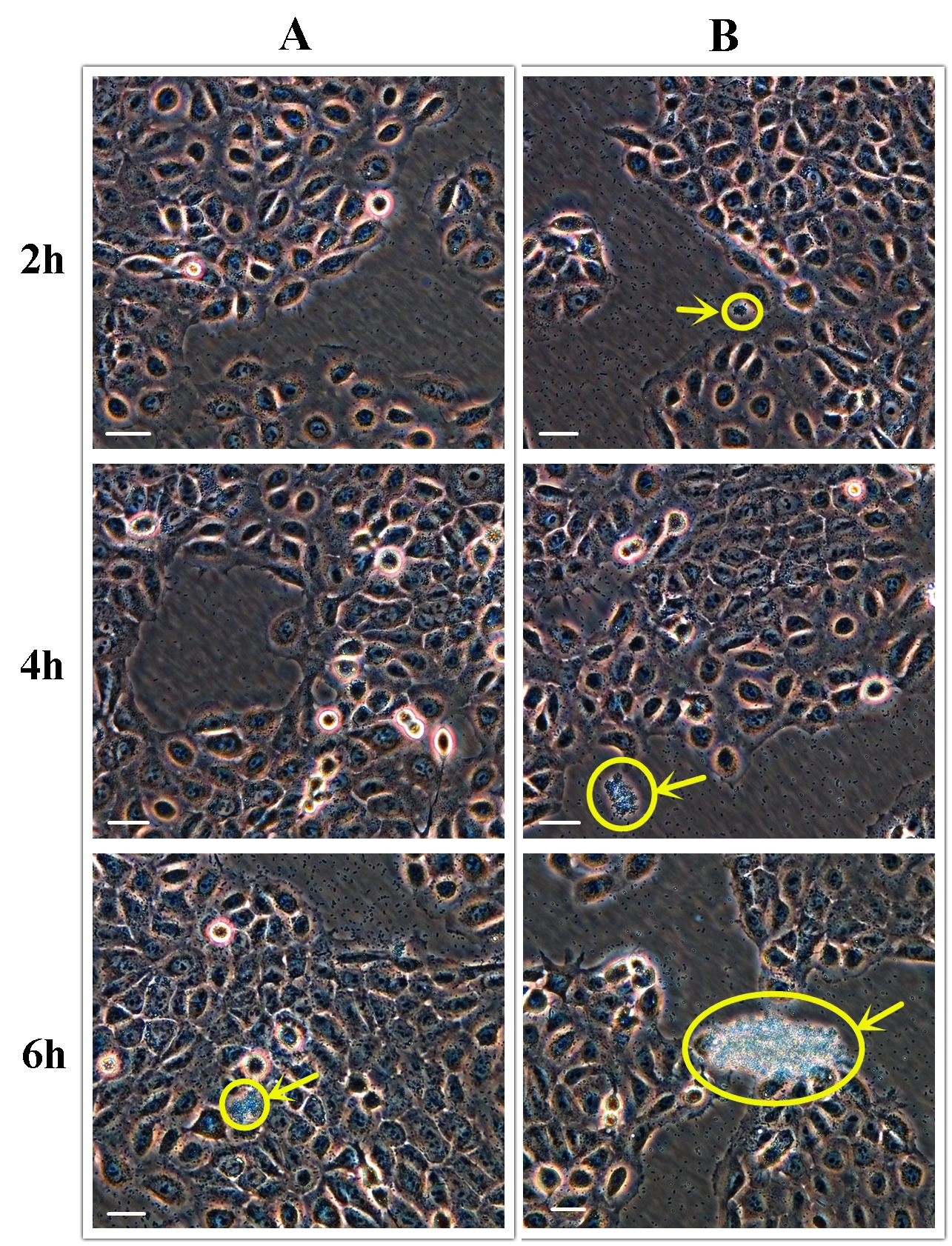


Fig. S3. **Microscopy observations of the effect of calcium on host-bacterial interaction.**

(A) Control group: the calcium final concentration was 0 mmol/L (with EDTA treatment). (B) Experimental group: the calcium supplementation final concentration was 1.4 mmol/L. Scale bar = 30 μm.

Calcium may promote Ab biofilm formation.
