# Supplementary material for: Effect of calcium on the interaction of Acinetobacter baumannii with human respiratory epithelial cells
Source: BMC Microbiol. 2019 Nov 27;19:264. doi: 10.1186/s12866-019-1643-z (PMC6880639; doi:10.1186/s12866-019-1643-z)
Supplement: Supplementary file 5 — Additional file 5: Table S2. Results of antimicrobial susceptibility testing of Ab used in this study. [file 12866_2019_1643_MOESM5_ESM.doc]

Additional file 5

Table S2. **Results of antimicrobial susceptibility testing of Ab used in this study.**

| Drug | Sensitivity * |
| --- | --- |
| Amoxicillin - Clavulanic acid | R |
| Piperacillin - Tazobactam | R |
| Cefoxitin | R |
| Ceftriaxone | R |
| cefepime | R |
| Aztreonam | R |
| Imipenem | R |
| Meropenem | R |
| Gentamicin | R |
| Tobramycin | R |
| Ciprofloxacin | R |
| Levofloxacin | R |
| Tigecycline | S |
| Sulfamethoxazole - Trimethoprim | R |
| Cefmetazole | R |
| Cefoperazone - Sulbactam | I |
| Ceftazidime | R |

*: susceptible, S; intermediate, I; resistance, R.

The results were detected by Vitek2-compact (Biomerieux Co., Ltd., France).
